# Transcriptome Sequencing of *Agave amaniensis* Reveals Shoot-Related Expression Patterns of *Expansin A* Genes in *Agave*

**DOI:** 10.3390/plants12102020

**Published:** 2023-05-18

**Authors:** Xuxia Wang, Xing Huang, Lisha Chen, Zhouli Xie, Shibei Tan, Xu Qin, Tao Chen, Yanlei Huang, Jingen Xi, Helong Chen, Kexian Yi

**Affiliations:** 1 Urban Construction College, Wuchang Shouyi University, Wuhan 430064, China; 2 Environment and Plant Protection Institute, Chinese Academy of Tropical Agricultural Sciences, Haikou 571101, China; 3 Quality Supervision, Inspection and Testing Center of Sisal and Products, Ministry of Agriculture and Rural Affairs, Zhanjiang 524022, China; 4 School of Life Sciences, Peking University, Beijing 100871, China; 5 Guangxi Subtropical Crops Research Institute, Nanning 530001, China; 6 College of Plant Science and Technology, Huazhong Agricultural University, Wuhan 430070, China; 7 Sanya Research Institute, Chinese Academy of Tropical Agricultural Sciences, Sanya 572025, China; 8 Key Laboratory of Integrated Pest Management on Tropical Crops, Ministry of Agriculture and Rural Affairs, Haikou 571101, China; 9 Hainan Key Laboratory for Monitoring and Control of Tropical Agricultural Pests, Haikou 571101, China

**Keywords:** transcriptome, *Agave amaniensis*, *expansin* genes, phylogeny, gene expression

## Abstract

*Agave* species are widely planted for fiber production. However, the molecular basis of agave fiber development has not been well understood. In this study, we performed a transcriptomic analysis in *A. amaniensi*, a well-known variety with high-quality fiber production. Approximately 43.87 million clean reads were obtained using Illumina sequencing. The de novo assembly produced 66,746 unigrams, 54% of which were annotated in a public database. In the Nr database, 21,490 unigenes of *A. amaniensis* were shown to be most closely related to *Asparagus officinalis*. Nine *expansin A* orthologs with full coding regions were obtained, which were named *EXP1a*, *EXP1b*, *EXP2*, *EXP3*, *EXP4a*, *EXP4b*, *EXP11*, *EXP12,* and *EXP13*. The maximum likelihood phylogenetic tree revealed the species-specific expansion of *expansin* genes in *Arabidopsis*, rice and agave. The expression analysis suggested the negative correlation between the expression of *expansin* genes and the leaf growth rate, except *AhEXP11*. Moreover, *expansin* genes were differentially affected by abiotic and biotic stresses. Notably, *AhEXP2* expression level was highly upgraded after the infection of *Phytophthora nicotiana*. Nutrient deficiency also influent *expansin* genes expression. Together, our research will benefit future studies related to fiber development, disease resistance and nutrient usage in agave.

## 1. Introduction

*Agave*, a perennial tropical hard leaf fiber crop, has been extensively cultivated as an economic crop for fiber production, food, and medicinal compounds in the tropic areas. It is also well known for its adaptation to the abiotic stresses such as xeric environments [1]. Its diverse usages and unique adaptation to environmental stresses make *Agave* an excellent candidate as a model crassulacean acid metabolism (CAM) crop [2]. It is composed of approximately 210 species around the world [3]. Among these, *A. amaniensis* Trel. & W. Nowell (1933) are famous for fibers production. A single plant can grow about 20 leaves, which are large, up to 150–200 cm long, and covered with a thick coating of waxy, pinkish-blue, thin fibers. *A. amaniensis* also has more than 2000 fiber bundles per leaf, twice as many as the common agave. Therefore, they are used to provide males to generate hybrid cultivars, such as H11648 ((*A. amaniensis* Trel. and Nowell × *A. angustifolia* Haw.) × *A. amaniensis*). H11648 with high fiber production and improved fiber-related traits becomes the main cultivar for sisal fiber production, especially in the tropical areas of Brazil, China and Africa [4,5].

To date, several studies related to agave fiber properties and some genes with fibrillar developmental correlations have been identified. For example, by comparing the transcriptomes with domesticated and wild agave species, a series of candidate genes regulating fructan, fiber, and stress response-related traits were identified in the varieties *A. tequilana*, *A. sisalana*, and *A. deserti*. Moreover, 12 *cellulose synthase* genes (*CesA*) in *Asparagus* genome and 38 *CesA* sequences from *A.* H11648, *A. americana*, *A. deserti* and *A. tequilana* were further identified [6]. *Cinnamyl Alcohol Dehydrogenase* (*CAD*) genes were also characterized in *A.* hybrid H11648, *A. deserti*, *A. tequilana*, *A. americana* and *A. angustifolia*, respectively. The expression analysis indicated that *CAD1*, *CAD2*, *CAD4* and *CAD6* were conservatively expressed, which may provide candidate targets for manipulation to improve the lignin properties [7]. However, the huge genomes and the long growth period of agave make it difficult to use genetic strategies to study the molecular mechanisms of fiber traits [8]. Therefore, the mechanisms of fiber development of agave are still not well understood. Due to the rapid development of transcriptome sequencing technology, we can use functional genomics to completely reveal genes related to fiber traits of agave species [9].

Plant cell walls are composed of cellulose, hemicellulose, lignin, and various other components [10]. The complex reticular structure determines the shape and function of cells along with growth and development, as well as their response to external environmental stimuli. During plant growth, cell shape and function are accompanied by a period of metamorphosis in which expansins play an important role [11]. Expansins are known to be important for non-enzymatic protein-wall loosening activity and to be involved in cell expansion and other developmental events during which cell wall modification occurs [12]. The expansin family contains a large amount of genes, and proteins encoded by this family are short: typically 225–300 amino acid residues in length. Generally, expansin family proteins can be divided into four subfamilies: α-expansin subfamily (generally named “EXPA”), β-expansin subfamily (named with “EXPB”), expansin-like A subfamily (“EXLA”), and expansin-like B subfamily (“EXLB”) [13], where dicotyledonous plants (*Arabidopsis thaliana*, poplar, grape, etc.) have more α-subfamily genes, while monocotyledonous plants (rice, wheat) have more β-subfamily expansin genes. In addition to its roles in cell wall metabolism such as cell elongation and expansion for regulating fiber elongation and fruit ripening [14,15], expansin is associated with multiple biotic and abiotic stress tolerance and plant–fungal interaction processes [16,17].Thus, we sequenced and assembled the leaf transcriptome of *A. amaniensis* based on Illumina sequencing to gain insight into gene expression levels related to fiber traits, and further performed a comparative analysis of expansin genes in six agave species. Together, these results provide new insights into the molecular underpinnings to study lignin traits in agave.

## 2. Results

### 2.1. Transcriptome Assembly and Annotation of A. amaniensis

**Figure 1:**
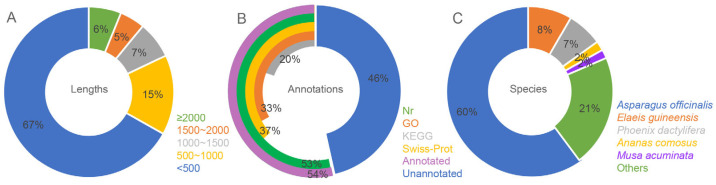
The length distribution (**A**) and annotation rates (**B**) of all unigenes and annotated species in Nr annotation (**C**).

**Figure 2:**
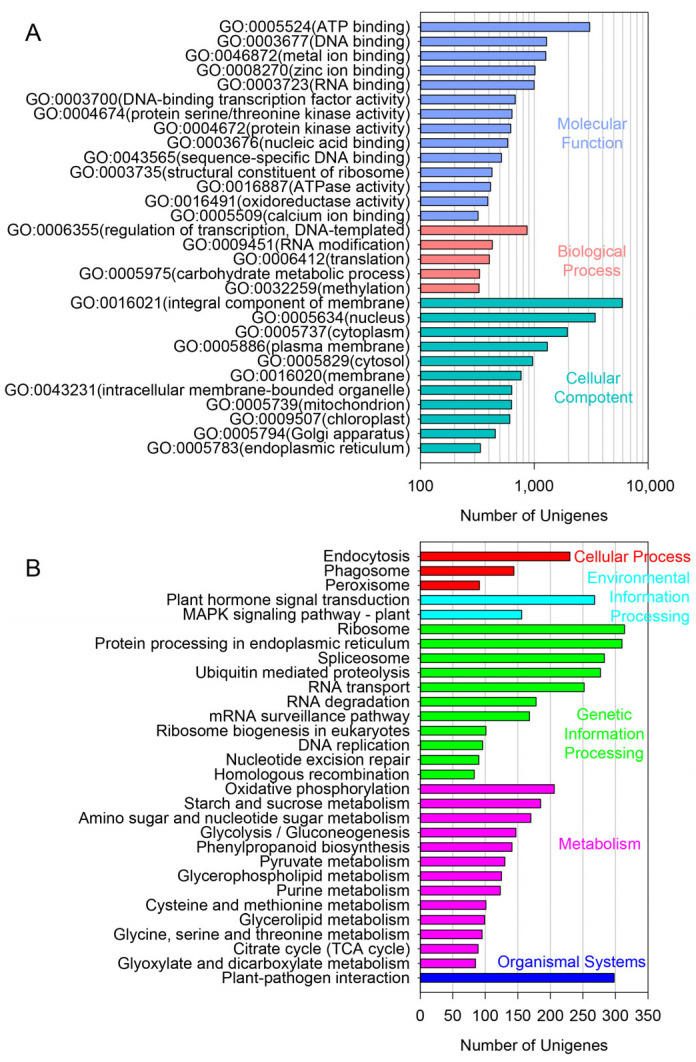
GO and KEGG annotation of all unigenes. (**A**), gene ontology (GO) annotation; (**B**), KEGG pathways analysis.

The leaf samples of *A. amaniensis* were used for transcriptome sequencing via the Illumina platform. The results showed that 43,865,662 clean reads were generated, with a total length of 6,028,970,833 bp and a GC content of 48.52%. The rates of base quality over 20 and 30 were 98.47% and 95.31%, respectively. A total of 66,764 unigenes were assembled from the clean data. The mean, median and N50 lengths were 654,347 and 1135, respectively. About 67% of the unigenes were shorter than 500 bp (Figure 1A, Table S1). More than half of these unigenes were annotated by at least one public database, including Nr (35,624), GO (22,041), KEGG (13,274) and Swiss-Prot (24,584) (Figure 1B). According to the Nr results, 21,490 unigenes of *A. amaniensis* were matched to orthologs in *Asparagus officinalis* (Figure 1C). After gene ontology (GO) annotation, the top abundant subcategory was “integral component of membrane”. Additionally, the other abundant subcategories were “nucleus”, “ATP binding”, “cytoplasm”, “plasma membrane”, “DNA binding”, “metal ion binding” and “zinc ion binding” (Figure 2A). The top eight KEGG pathways were “Ribosome”, “Protein processing in endoplasmic reticulum”, “Plant-pathogen interaction”, “Spliceosome”, “Ubiquitin mediated proteolysis”, “Plant hormone signal transduction”, “RNA transport” and “Endocytosis” (Figure 2B).

### 2.2. Identification and Cloning of Expansin A Genes in Agave Species

In order to identify *expansin A* genes in agave species, expansin A proteins from *Arabidopsis* (26) and rice (33) were first selected to search orthologs in the *Asparagus* genome. In this way, 17 *expansin* genes were generated (Table S2). After these genes were combined, the 76 proteins were used to identify orthologs in the transcriptome datasets of six agave species, including *A. amaniensis*, *A. deserti*, *A. tequilana*, *A. americana*, *A. angustifolia* and *A*. H11648. Thirty orthologs with full coding regions were obtained and named as *EXP1a*, *EXP1b*, *EXP2*, *EXP3*, *EXP4a*, *EXP4b*, *EXP11*, *EXP12*, and *EXP13* based on the sequence similarity. These full-length *EXP* genes were further used to amplify the gaps or genes in agave species for each group, which finally generated a total of 54 *expansin* genes (Table S3. Each agave species has nine genes).

### 2.3. Phylogeny of Expansin Genes

**Figure 3:**
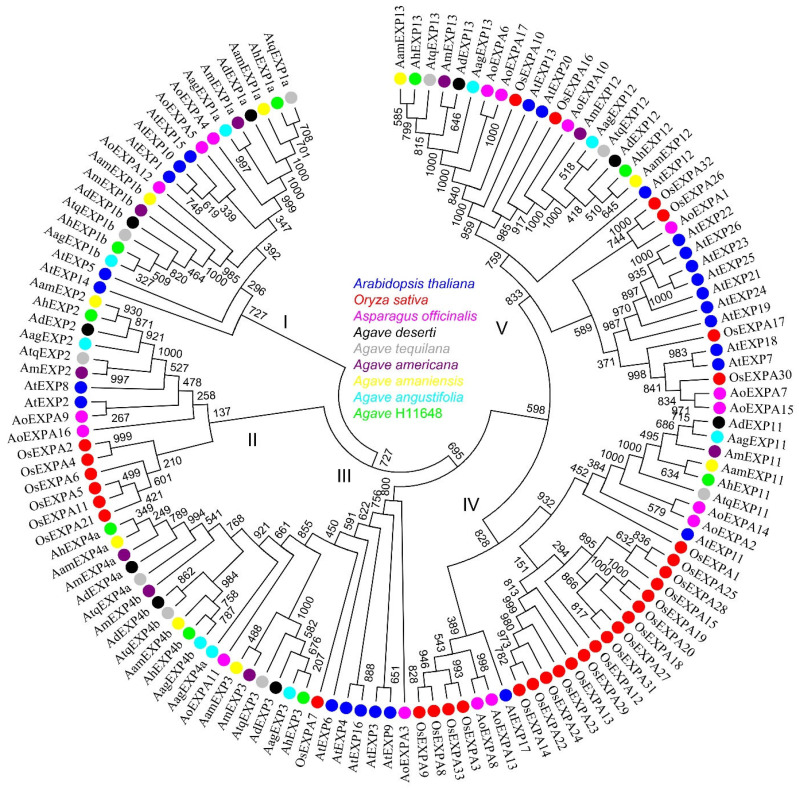
Phylogenetic relationship of *expansin A* family. The protein sequences of *Arabidopsis thaliana*, *Oryza sativa*, *Asparagus officinalis*, *A. deserti*, *A. tequilana*, *A. americana*, *A. amaniensis*, *A. angustifolia* and *A*. H11648 are shown in blue, red, pink, black, gray, purple, yellow, light blue and green, respectively.

The 130 *expansin A* genes (mentioned in Section 2.2) from Arabidopsis, rice, asparagus and six agave species were selected and used for phylogenetic analysis. After phylogenetic analysis, these selected genes were clustered into five groups (Figure 3). Typically, the *expansin* genes of the six agave species were grouped together, with equal numbers present in each group. Each agave species had two genes in Group I, one gene in Group II, three genes in Group III, one gene in Group IV, and two genes in Group V, the numbers of which were much smaller than those in Arabidopsis. Because of that, Arabidopsis contained five, two, five, two, and twelve expansin genes in Group I–V, respectively. In addition, more agave sequences were present in Group III, while more rice sequences were present in Groups II and IV, with the most occurring in Group IV.

### 2.4. Expression Patterns of Expansin A Genes in A. H11648

**Figure 4:**
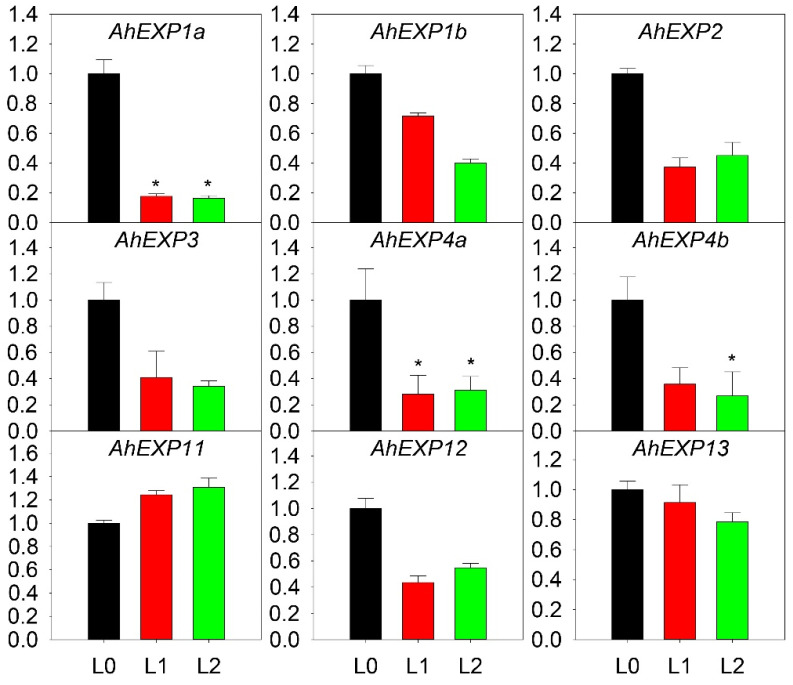
Relative expression quantities of agave *expansin* genes in different leaf developmental stages. Each bar chart (L0 to L2) was formed as the order of samples shoot, unexpanded leaf and expanded leaf (*X*-axis) for their quantitative results (*Y*-axis). The error bar represented the standard error. The asterisks (*) indicated that the expression level of the genes decreased more than threefold, and the asterisks marked the significant differences when compared with the control under *p* value of 0.05. The expression values of each gene in L0 were normalized as 1.

In order to detect expression patterns of *expansin* A genes, *A.* H11648 was selected for further Quantitative Real-Time PCR (qRT-PCR) analysis. Expression patterns were estimated at different leaf developmental stages (Figure 4). The result revealed that these nine genes had different expression patterns. In shoot, all nine *expansin* genes had high expression levels, with only *AhEXP11* being slightly lower. In unexpanded leaf, *AhEXP11*, *AhEXP1b* and *AhEXP13* had high expression levels. *AhEXP11* and *AhEXP13* also had high expression levels in expanded leaf. In other words, the expression levels of *AhEXP11* and *AhEXP13* were high in the three leaf developmental stages. The difference was that the expression level of *AhEXP11* increased with development, whereas *AhEXP13* decreased but still maintained a high level. The expression patterns of *AhEXP1a* and *AhEXP4a* were similar, with high expression levels during the shooting stages but a drop in the unexpanded leaf and expanded leaf. In summary, there was a downward trend in expression of the agave *expansin* genes except for *AhEXP11*. The most important thing to mention was that the expression of the *AhEXP1a* and *AhEXP4a* decreased significantly with the leaf growth.

### 2.5. Expression of Expansin A Genes under Abiotic and Biotic Stresses

**Figure 5:**
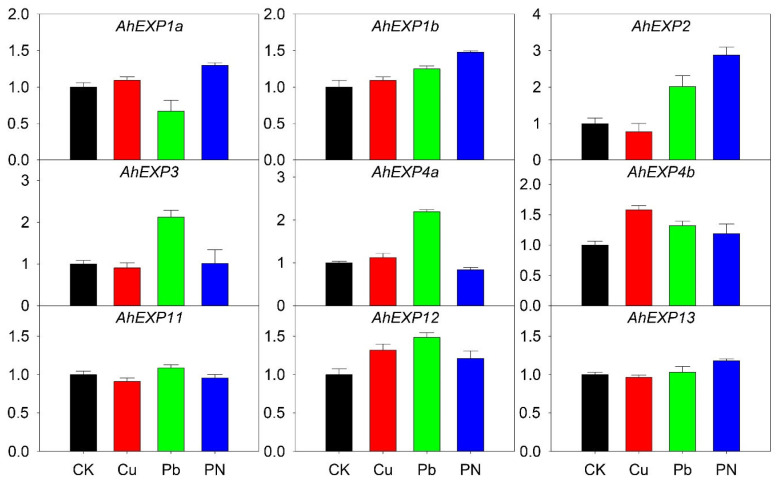
Relative expression quantities of agave *expansin* genes under abiotic and biotic stresses, including copper (Cu) and lead (Pb) treatments and *Phytophthora nicotianae* Breda infection (PN). The *X*-axis showed the different treatments. *Y*-axis showed their quantitative results. The error bar represented the standard error. The expression values of control (CK) were normalized as 1.

*A*. H11648 is often infected by pathogenic microorganisms during its growth and development, while *A*. H11648 is more tolerant to Cu and Pb stresses. Therefore, Cu and Pb stresses as abiotic stresses and one biotic stress infected with *P. nicotianae* Breda were evaluated to assess the expression of the *expansin* genes in *A*. H11684 leaves, respectively. Seven genes were expressed differentially under one of these stresses, i.e., *AhEXP4b* and *AhEXP12* under Cu stress, *AhEXP1b*, *AhEXP2*, *AhEXP3*, *AhEXP4a*, *AhEXP4b* and *AhEXP12* under Pb stress. The genes expression level of *AhEXP1a*, *AhEXP1b*, *AhEXP2*, *AhEXP12* and *AhEXP13* were highly upgraded after infected with *P. nicotiana*, especially *AhEXP2*. The expression levels of genes *AhEXP11* and *AhEXP13* did not change much under control, abiotic and biotic stresses stress, and both were at very high levels (Figure 5).

### 2.6. Expression of Expansin A Genes under Nutrient Deficiency

**Figure 6:**
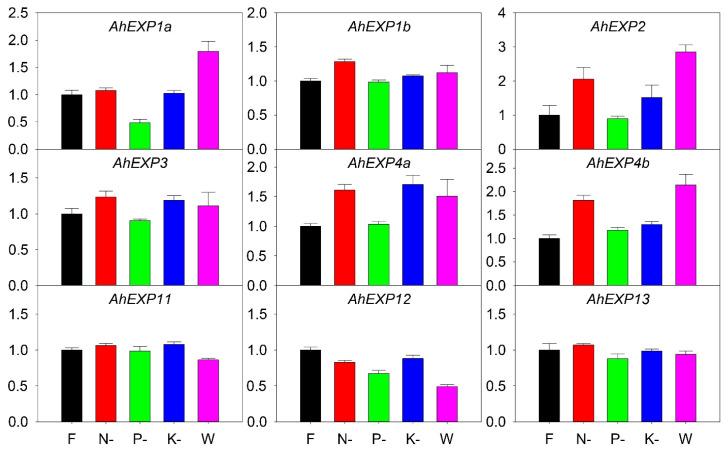
Relative expression quantities of agave *expansin* genes under nutrient deficiency, including full- (F), nitrogen free- (N-), phosphorus free- (P-), potassium free- (K-) Hoagland nutrient solutions and water (W). The *x*-axis shows the different treatments. The *Y*-axis was their quantitative results. The error bar represented the standard error. The expression values of F were set as control and normalized as 1.

Further nutrient deficiency treatments were carried out to study the expression profiling of expansin genes. The full Hoagland nutrient solution was used as control (F). Hoagland nutrient solutions without nitrogen (N-), phosphorus (P-), potassium (K-) and water (W) control were used to test the impact of nutritional factors. The results indicated that *AhEXP1b*, *AhEXP2*, *AhEXP3*, *AhEXP4a* and *AhEXP4b* were sensitive to nutrient nitrogen, while *AhEXP1a*, *AhEXP11* and *AhEXP13* were less sensitive (Figure 6). Most *expansin* genes were less sensitive to nutrient phosphorus, including *AhEXP1b*, *AhEXP2*, *AhEXP11*, *AhEXP4a*, *AhEXP4b*, *AhEXP11* and *AhEXP13*, but the expression of *AhEXP1a* was decreased. The expressions of *AhEXP4a* were upregulated under K-. Additionally, *AhEXP1a*, *AhEXP2* and *AhEXP4b* showed upregulated expressions in W, but expression of *AhEXP12* was decreased.

## 3. Discussion

### 3.1. Characterization of A. amaniensis Transcriptome

Over the past decade, RNA sequencing (RNA-seq) has become an indispensable tool for transcriptome-wide analysis of differential gene expression and differential splicing of mRNAs [18]. Compared to DNA microarray-based methods, RNA-Seq offers less background noise and a greater dynamic range for detection [19]. Most importantly, RNA-Seq directly reveals sequence identity, which is crucial for analysis of unknown genes and novel transcript isoforms [20]. Because of the large genomes of agave, transcriptome analysis becomes an efficient method for gene mining. However, the expressed sequence tags (EST) sequences of agave species were limited and rare in the NCBI database, which influences the understanding of the sisal genome, transcriptome, and the agave fiber development [5]. Although transcriptomes of *A.* H11648 [5], *A. angustifolia* [7], *A. tequilana*, *A. sisalana*, and *A. deserti* [6] were revealed by Illumina Sequencing, and a series of candidate genes were predicted to affect fructans, fiber traits and stress response-related traits, genetic sequence, and transcriptome information about *A. amaniensis* have not been submitted to GenBank, leaving a knowledge gap in molecular basis.

Therefore, we assembled the transcriptome of *A. amaniensis* with 66,746 unigenes. According to this new assembly, the unigenes number was much the same as that in *A. angustifolia* (66,314) but far less than *A.* H11648 (148,046). Because transcriptome sequencing requires high-quality samples, the discrepancy of identified unigenes in agave varieties might be caused by differences in sample collection, sample quality, and transcriptome assembly methods [21]. Together, 18% of the unigenes had sequence lengths over 1000 bp, and 15% of the sequences were in the 500–1000 bp range (Table S1). The longer sequence length provides a greater opportunity to identify homologs in public databases. Therefore, about 54% of unigenes have homologs in public databases (Figure 1), which facilitated the prediction of agave gene functions.

### 3.2. Candidate Expansin A Genes in Shoot Development of Agave

Expansins are known to be involved in the loosening of plant cell walls, which allows for cell expansion and elongation. This process is important for growth and development in many plants, including those used for fiber production such as cotton [22], flax [23] and ramie [15].

In these crops, expansins are believed to play a role in fiber development by promoting the elongation and thickening of individual cells, leading to longer and stronger fibers. As an important fiber crop in tropical area, the quality and yield of fiber production in agave are the important traits for agave cultivation. One of the agave varieties, *A. amaniensis*, has been used for phytosteroid production for a long time [24]. This variety is also known for its large leaves, which can grow up to 150–200 cm in length. These leaves are typically harvested for their strong and durable fibers, which are composed of more than 2000 fibrous bundles per leaf. However, the plant’s large size and slow growth rate make it less suitable for commercial fiber production. Nowadays, *A. amaniensis* is usually used as male parent to generate the famous *Agave* hybrid cultivar H11648. Thus, we reasoned that *A. amaniensis* might be rich in genes related to fiber development.

After transcriptome sequencing in *A. amaniensis*, we retrieved *expansin* genes. As a result, we identified 9 *expansin* genes in *A. amaniensis* and generated a total of 54 *expansin* genes in the 6 agave species (Table S3. Each agave species has 9 genes). The expansin gene family size in asparagus (17) (Table S2) and agave (9) is smaller than that in Arabidopsis (26) and rice (33). The smaller size of agave *expansin* genes might be due to tissue-specific expression or the limitation of RNA-Seq in our study. Although a series of saponin-related [25] and fructan-related [26] research studies have been reported, few reports were related to fiber development. Therefore, these *expansin* genes we identified can serve as guidance for further studies on agave fiber development. The phylogenetic analysis indicated that *expansin* genes have a conserved evolutionary pattern among different species (Figure 3), regardless of some species-specific expansion of *expansin* genes, such as Arabidopsis in group V and rice in group II and IV.

In shoot, all nine *expansin* genes had high expression levels, but with the leaf growth, most of the expression genes decreased significantly, especially *AhEXP1a* and *AhEXP4a*. Results took on low expression in leaf samples (Figure 4). The negative correlation between the expression of *expansin* genes and the leaf elongation rate is contrary to expansin’s molecular function. Considering the species-specific expression of *expansin*, we reasoned that the decreased expression level of *expansin* during leaf growth is due to their tissue-specific expression [27] or transcriptional regulations [5], which needs further study in the future.

Abiotic and biotic stresses, along with artificial selection, determine the economic traits for agave domestication. In our study, Cu, Pb and fungus’ effects on *expansin* expression are evaluated. Two, seven, and four *expansin* genes were significantly up- or down-regulated under the three stresses, especially *AhEXP2*, *AhEXP3* and *AhEXP4a* (Figure 5), indicating *expansin* genes might have different response patterns to abiotic/biotic stresses. Sisal zebra mosaic disease caused by *P. nicotiana* is one of the most serious diseases in sisal production. Notably, after infection with *P. nicotiana*, *AhEXP2* expression level was highly upgraded, implying it might be involved in the synthesis of secondary metabolites associated with disease resistances. This is mainly due to the different sensing and regulatory networks upstream of various antioxidant enzymes, which are generally produced in plants to clean reactive oxygen species triggered by stress [28]. *Expansin* genes are also involved in N, P and K responses. In our cases, *AhEXP2*, *AhEX4a* and *AhEXP4b* are involved in the N response, but have minimal responses to P. However, *AhEX1a* was down-regulated under P deficit condition. Although some reports in *Brassica napus* examined the crosstalk among the three nutrients [29], more efforts are needed to reveal the interactions among N, P and K nutrients to finally facilitate the application of fertilizers in agave.

## 4. Materials and Methods

### 4.1. Plant Materials and RNA Extraction

*A. amaniensis* and *A.* H11648 were planted and normally managed in the Wenchang experimental field (19.00° N, 110.33° E) of the Environment and Plant Protection Institute, Chinese Academy of Tropical Agricultural Sciences. Shoots, unexpanded leaves, and expanded leaves were collected separately from two-year-old plants at different stages of development [30].

Leaves treated with abiotic and biotic stress were conducted using one-year-old plants, which has been described in our previous study [30]. *A.* H11648 is tolerant to stress from heavy metals, such as copper and lead [31,32]. Thus, in this study, we utilized CuSO_4_ solution and Pb(NO_3_)_2_ solution watering as the abiotic stresses. The CuSO_4_ solution concentration was 1 g/Kg (heavy metal salt/soil) and the Pb(NO_3_)_2_ solution concentration was 1.3 g/Kg (heavy metal salt/soil). About two weeks later, the leaves of treated *A.* H11648 plants started curling. At this point, the leaves were collected as test samples. Zebra disease is a serious threat to the main cultivar *A.* H11648 worldwide. The pathogen has been identified as *P. nicotianae* Breda [33]. So, in our study, *P. nicotianae* Breda strain was inoculated on *A.* H11648 leaves as the biotic stress. The strain of *P. nicotianae* Breda used for inoculation was isolated earlier from our laboratory. The young parts of the sisal leaves were selected for inoculation. The leaves were firstly disinfected with alcohol, then washed with sterilized water, and afterwards they were dried and inoculated. The control group was inoculated without *P. nicotianae* Breda.

As high levels of irrigation and fertilizer are required to sustain high yields of agaves species, the full (F), nitrogen free (N-), phosphorus free (P-), potassium free (K-) Hoagland nutrient solutions and water (W), were used to irrigate *A.* H11648 to study the expression pattern of agave *expansin* genes under nutrient deficiency conditions [30].

The tests for each set of treated and untreated leaves were repeated three times with different individual plants as biological replicates. The collected leaves were immediately frozen into liquid nitrogen. The total RNA was extracted according to the manufacturer’s protocol using the RNA extraction kit (Tiangen Biomart, Beijing, China). The total RNA samples were immediately placed into liquid nitrogen and stored at −80 °C.

### 4.2. Transcriptome Sequencing, Assembly and Annotation

The total RNA samples of *A. amaniensis* were used for library construction and Illumina sequencing (Genoseq Technology Co., Ltd. Wuhan, China) [34]. After quality detection, total RNA was used for mRNA extraction. mRNA was collected using the magnetic bead method and then fragmented using Illumina TruSeq RNA kit (San Diego, CA, USA). The first cDNA was synthesized using mRNA and reverse transcriptase M-MuLV with random hexamer primers as guidance. Additionally, the second-strand cDNA was synthesized using RNase H and DNA polymerase I. Next, the double-strand cDNA fragments were modified with a single ‘A’ bases and added with adaptors. Additionally, the cDNA library was constructed after gel purification and PCR amplification. Sequencing of the cDNA library was performed with the HiSeq platform of Illumina, which generated paired-end raw reads of 150 bp in length.

We submitted the raw reads to the public Sequence Read Archive (SRA) database and the accession number was PRJNA917773 [35]. Clean reads were obtained by filtering the adaptors and low-quality sequences using the software Cutadapt (version 4.4) and Trimmomatic (version 0.38), respectively [36,37,38]. The assembly of de novo transcriptome of *A. amaniensis* was carried out with the Trinity software (version 2.8.4), which was annotated according to public databases. Four public databases, Nr (NCBI non-redundant protein database) [39], GO (the Gene Ontology) [40], KEGG (the Kyoto Encyclopedia of Genes and Genomes) [41,42], and the Swiss-Prot database [43], were selected for functional annotation. The Blastx method was selected to search orthologs in the four public databases with a cut-off E value of 10^−5^ [5].

### 4.3. Characterization and Phylogeny of Expansin Genes

The expansin A proteins from model plants *A. thaliana* (26) and *Oryza sativa* (33) were selected to search orthologs in asparagus genome using the Tblastn method with a cut-off E value of 10^−5^ [15,44]. The generated *expansin* genes were used to identify orthologs in the transcriptome datasets of six agave species, which were previously published. Additionally, the transcriptome data from the species of *A. deserti* [45], *A. tequilana*, *A. americana*, *A. amaniensis*, *A. angustifolia* and *A*. H11648 [5,30,46]. *Asparagus officinalis* (*Asparagoideae*) and agave species (*Agavoideae*) all belonged to *Asparagaceae* with close phylogenetic position, so *Asparagus officinalis* was chosen as the reference species [47,48].

The full-length *expansin* genes of the nine species (Arabidopsis, rice, *A. officinalis*, *A. deserti* [45], *A. tequilana*, *A. americana*, *A. amaniensis*, *A. angustifolia* and A. H11648) were aligned using the ClustalX method for phylogenetic analysis. The maximum likelihood tree with a bootstrap value of 1000 trials was constructed using the MEGA 7.0 software [49]. The conserved amino acid residues of these protein sequences of the nine species were further detected by alignment using the DNAMAN software (version 9.0.1.116).

### 4.4. Expression Patterns of Agave Expansin Genes

The expression patterns of agave *expansin* genes were tested using qRT-PCR at the developmental stages of shoot, non-expanding leaf, and expanding leaf. The total RNA was extracted using the RNA extraction kit according to the manufacturer’s protocol (Tiangen Biomart, Beijing, China), and then digested using DNase I enzymes. All the RNA samples were reverse transcribed to the cDNA using the Reverse Transcription System (Promega, Madison, WI, USA). The qRT-PCR reaction system was 20 µL, including a 1 µL cDNA template, 10 µL TransStart Tip Green qPCR Supermix (Transgen Biotech, Beijing, China), 0.5 µL forward primer (10 µM), 0.5 µL reverse primer (10 µM), 0.4 µL Passive Reference Dye (50×) (Transgen Biotech, Beijing, China), and 7.6 µL ddH_2_O. Technical replicates were performed three times for each sample. Nine pairs of specific primers for agave *expansin* genes and one pair for reference gene *phosphatase 2A* (*PP2A*) as an endogenous control were synthesized; these were designed using Primer 3 software (version 0.4.0) (Table 1) [50]. The qRT-PCR procedure was conducted with the program of an initial stage (94 °C, 30 s), 40 cycles in a stage of 94 °C for 5 s and 60 °C for 30 s and a final dissociation stage by a QuantStudio 6 Flex Real-Time PCR System (Thermo Fisher Scientific, Waltham, MA, USA). The ∆∆Ct method was used to calculate relative expression levels with *PP2A* as an endogenous reference gene [51,52]. The data were analyzed according to variance and plotted.

## Figures and Tables

**Table 1:** Primers for qRT-PCR analysis.

Genes	Forward Primers	Reverse Primers	Product Length (bp)
*AhEXP1a*	CAGTACAGGGCTGGGATTGT	CTGGCCATTGAGGTAGGTGT	234
*AhEXP1b*	AACAATGGGAGGAGCTTGTG	GCATTGTTTGGGAGAGCATT	216
*AhEXP2*	GCACCTTCAGTGGCTTCTTC	AGAGATTGCCGTACCCACAG	181
*AhEXP3*	ATGGCTCCCCTTGCTATTCT	TGGCTGTACAAATTGCCGTA	179
*AhEXP4a*	TTCTCTCTCTCTGGCCCTCA	AAATAACGCCGTGCTTAACG	226
*AhEXP4b*	GGGGACATCACGAAGGTCTA	AAGTTTTTCCCGGTGAAGGT	209
*AhEXP11*	GTGCGGTCAGTGCTACAAGA	GCATGGAACCCTTTGGTAGA	241
*AhEXP12*	GCCTGCCTTCATCTCAAAAC	TTCTTACCGTACCCCGACTG	212
*AhEXP13*	CTGGAGATGTGACGGCTGTA	TTGGGTGCAACGTTGTAAGA	172
*PP2A*	CCTCCTCCTCCTTCGGTTTG	GCCATGAATGTCACCGCAGA	235

## Data Availability

All data are contained within the article or Supplementary Materials.

## Supplementary Materials

The following supporting information can be downloaded at: https://www.mdpi.com/article/10.3390/plants12102020/s1, Table S1: The annotation of all unigenes in Nr, GO, KEGG and Swiss-Prot databases, Table S2: The *expansin* gene family in asparagus, Table S3: Details of *expansin* genes in six agave species.

## References

[1] Borland A.M., Griffiths H., Hartwell J., Smith J.A. (2009). Exploiting the potential of plants with crassulacean acid metabolism for bioenergy production on marginal lands. J. Exp. Bot..

[2] Stewart J.R. (2015). *Agave* as a model CAM crop system for a warming and drying world. Front. Plant Sci..

[3] Lopez-Romero J.C., Ayala-Zavala J.F., Gonzalez-Aguilar G.A., Pena-Ramos E.A., Gonzalez-Rios H. (2018). Biological activities of *Agave* by-products and their possible applications in food and pharmaceuticals. J. Sci. Food Agric..

[4] Xu B., Tan S., Qin X., Huang X., Xi J., Chen H., Qin J., Chen T., Yi K. (2022). The complete chloroplast genome of *Agave amaniensis* (*Asparagales*: *Asparagaceae*: *Agavoideae*). Mitochondrial DNA B Resour..

[5] Huang X., Xiao M., Xi J., He C., Zheng J., Chen H., Gao J., Zhang S., Wu W., Liang Y. (2019). De Novo transcriptome assembly of Agave H11648 by Illumina Sequencing and identification of cellulose synthase genes in *Agave* species. Genes.

[6] Huang X., Wang B., Xi J., Zhang Y., He C., Zheng J., Gao J., Chen H., Zhang S., Wu W. (2018). Transcriptome comparison reveals distinct selection patterns in domesticated and wild *Agave* species, the important CAM plants. Int. J. Genom..

[7] Huang X., Xu B., Tan S., Huang Y., Xi J., Qin X., Chen T., Chen H., Yang X., Yi K. (2022). Transcriptome sequencing of *Agave angustifolia* reveals conservation and diversification in the expression of cinnamyl alcohol dehydrogenase genes in *Agave* species. Agriculture.

[8] Robert M.L., Lim K.Y., Hanson L., Sanchez-Teyer F., Bennett M.D., Leitch A.R., Leitch I.J. (2008). Wild and agronomically important *Agave* species (*Asparagaceae*) show proportional increases in chromosome number, genome size, and genetic markers with increasing ploidy. Bot. J. Linn. Soc..

[9] Schuster S.C. (2008). Next-generation sequencing transforms today’s biology. Nat. Methods.

[10] Wallace I.S., Anderson C.T. (2012). Small molecule probes for plant cell wall polysaccharide imaging. Front. Plant Sci..

[11] Cosgrove D.J. (2015). Plant expansins: Diversity and interactions with plant cell walls. Curr. Opin. Plant Biol..

[12] Sampedro J., Cosgrove D.J. (2005). The expansin superfamily. Genome Biol..

[13] Kende H., Bradford K., Brummell D., Cho H.T., Cosgrove D., Fleming A., Gehring C., Lee Y., McQueen-Mason S., Rose J. (2004). Nomenclature for members of the expansin superfamily of genes and proteins. Plant Mol. Biol..

[14] Wang Y., Deng L., Meng J., Niu L., Pan L., Lu Z., Cui G., Wang Z., Zeng W. (2021). Transcriptomic and metabolic analyses reveal the mechanism of ethylene production in stony hard peach fruit during cold storage. Int. J. Mol. Sci..

[15] Chen J., Dai L., Wang B., Liu L., Peng D. (2015). Cloning of expansin genes in ramie (*Boehmeria nivea* L.) based on universal fast walking. Gene.

[16] Abuqamar S., Ajeb S., Sham A., Enan M.R., Iratni R. (2013). A mutation in the expansin-like A2 gene enhances resistance to necrotrophic fungi and hypersensitivity to abiotic stress in *Arabidopsis thaliana*. Mol. Plant Pathol..

[17] Morales-Quintana L., Barrera A., Hereme R., Jara K., Rivera-Mora C., Valenzuela-Riffo F., Gundel P.E., Pollmann S., Ramos P. (2021). Molecular and structural characterization of expansins modulated by fungal endophytes in the *Antarctic Colobanthus quitensis* (Kunth) Bartl. Exposed to drought stress. Plant Physiol. Biochem..

[18] Li Z., Xu Y. (2022). Bulk segregation analysis in the NGS era: A review of its teenage years. Plant J..

[19] Hrdlickova R., Toloue M., Tian B. (2017). RNA-Seq methods for transcriptome analysis. Wiley Interdiscip. Rev. RNA.

[20] Ura H., Togi S., Niida Y. (2022). A comparison of mRNA sequencing (RNA-Seq) library preparation methods for transcriptome analysis. BMC Genomics.

[21] Sadamoto H., Takahashi H., Okada T., Kenmoku H., Toyota M., Asakawa Y. (2012). De novo sequencing and transcriptome analysis of the central nervous system of mollusc Lymnaea stagnalis by deep RNA sequencing. PLoS ONE.

[22] Li Y., Tu L., Pettolino F.A., Ji S., Hao J., Yuan D., Deng F., Tan J., Hu H., Wang Q. (2016). GbEXPATR, a species-specific expansin, enhances cotton fibre elongation through cell wall restructuring. Plant Biotechnol. J..

[23] Roach M.J., Deyholos M.K. (2008). Microarray analysis of developing flax hypocotyls identifies novel transcripts correlated with specific stages of phloem fibre differentiation. Ann. Bot..

[24] Indrayanto G., Rahayu L., Rahman A., Noeraeni P.E. (1993). Effect of calcium, strontium, and magnesium ions on the formation of *Phytosteroids* in callus cultures of *Agave amaniensis*. Planta Med..

[25] de Oliveira J., Botura M.B., Dos S.J., Argolo D.S., Da S.V., Da S.G., de Lima H.G., Braz F.R., Vieira I., Branco A. (2019). Saponin-rich fraction from *Agave sisalana*: Effect against malignant astrocytic cells and its chemical characterisation by ESI-MS/MS. Nat. Prod. Res..

[26] Corbin K.R., Byrt C.S., Bauer S., DeBolt S., Chambers D., Holtum J.A., Karem G., Henderson M., Lahnstein J., Beahan C.T. (2015). Prospecting for energy-rich renewable raw materials: Agave leaf case study. PLoS ONE.

[27] Fan N., Xu Q., Yang Z., Zhuang L., Yu J., Huang B. (2023). Identification of expansin genes as promoting or repressing factors for leaf elongation in tall fescue. Physiol. Plant.

[28] Zhang H., Zhu J., Gong Z., Zhu J.K. (2022). Abiotic stress responses in plants. Nat. Rev. Genet..

[29] Nath M., Tuteja N. (2016). NPKS uptake, sensing, and signaling and miRNAs in plant nutrient stress. Protoplasma.

[30] Tan S., Liang Y., Huang Y., Xi J., Huang X., Yang X., Yi K. (2022). Phylogeny and expression atlas of the nitrate transporter 1/peptide transporter family in *Agave*. Plants.

[31] Chen L., Zhang L., Li F.Y., Guo B., Li X., Liao X., Qi Z. (2007). A primary research on sisal’s uptake property and the accumulation rule to Pb ions. J. Agro Environ. Sci..

[32] Li F.Y., Zhang L., Li X., Guo B., Chen L., Qi Z. (2006). Sisal tolerance of cupreous and its accumulation preliminary explore. Chin. Agric. Sci. Bull..

[33] Gao J., Luo P., Guo C., Li J., Liu Q., Chen H., Zhang S., Zheng J., Jiang C., Dai Z. (2012). AFLP analysis and zebra disease resistance identification of 40 sisal genotypes in China. Mol. Biol. Rep..

[34] Huang X., Chen J., Bao Y., Liu L., Jiang H., An X., Dai L., Wang B., Peng D. (2014). Transcript profiling reveals auxin and cytokinin signaling pathways and transcription regulation during in vitro organogenesis of Ramie (*Boehmeria nivea* L. Gaud). PLoS ONE.

[35] Katz K., Shutov O., Lapoint R., Kimelman M., Brister J.R., O’Sullivan C. (2022). The sequence read archive: A decade more of explosive growth. Nucleic Acids Res..

[36] Martin M. (2011). Cutadapt removes adapter sequences from high-throughput sequencing reads. EMBnet J..

[37] Grabherr M.G., Haas B.J., Yassour M., Levin J.Z., Thompson D.A., Amit I., Adiconis X., Fan L., Raychowdhury R., Zeng Q. (2011). Full-length transcriptome assembly from RNA-Seq data without a reference genome. Nat. Biotechnol..

[38] Bolger A.M., Lohse M., Usadel B. (2014). Trimmomatic: A flexible trimmer for Illumina sequence data. Bioinformatics.

[39] Pruitt K.D., Tatusova T., Maglott D.R. (2007). NCBI reference sequences (RefSeq): A curated non-redundant sequence database of genomes, transcripts and proteins. Nucleic Acids Res..

[40] Ashburner M., Ball C.A., Blake J.A., Botstein D., Butler H., Cherry J.M., Davis A.P., Dolinski K., Dwight S.S., Eppig J.T. (2000). Gene ontology: Tool for the unification of biology. The Gene Ontology Consortium. Nat. Genet..

[41] Kanehisa M., Goto S. (2000). KEGG: Kyoto encyclopedia of genes and genomes. Nucleic Acids Res..

[42] Ogata H., Goto S., Sato K., Fujibuchi W., Bono H., Kanehisa M. (1999). KEGG: Kyoto encyclopedia of genes and genomes. Nucleic Acids Res..

[43] Bairoch A., Apweiler R. (1999). The SWISS-PROT protein sequence data bank and its supplement TrEMBL in 1999. Nucleic Acids Res..

[44] Mount D.W. (2007). Using the basic local alignment search tool (BLAST). CSH Protoc..

[45] Gross S.M., Martin J.A., Simpson J., Abraham-Juarez M.J., Wang Z., Visel A. (2013). De novo transcriptome assembly of drought tolerant CAM plants, *Agave deserti* and *Agave tequilana*. BMC Genom..

[46] Abraham P.E., Yin H., Borland A.M., Weighill D., Lim S.D., De Paoli H.C., Engle N., Jones P.C., Agh R., Weston D.J. (2016). Transcript, protein and metabolite temporal dynamics in the CAM plant *Agave*. Nat. Plants.

[47] Harkess A., Zhou J., Xu C., Bowers J.E., Van der Hulst R., Ayyampalayam S., Mercati F., Riccardi P., McKain M.R., Kakrana A. (2017). The asparagus genome sheds light on the origin and evolution of a young Y chromosome. Nat. Commun..

[48] McKain M.R., Wickett N., Zhang Y., Ayyampalayam S., McCombie W.R., Chase M.W., Pires J.C., DePamphilis C.W., Leebens-Mack J. (2012). Phylogenomic analysis of transcriptome data elucidates co-occurrence of a paleopolyploid event and the origin of bimodal karyotypes in *Agavoideae* (*Asparagaceae*). Am. J. Bot..

[49] Kumar S., Stecher G., Tamura K. (2016). MEGA7: Molecular evolutionary genetics analysis Version 7.0 for bigger datasets. Mol. Biol. Evol..

[50] Untergasser A., Cutcutache I., Koressaar T., Ye J., Faircloth B.C., Remm M., Rozen S.G. (2012). Primer3--new capabilities and interfaces. Nucleic Acids Res..

[51] Livak K.J., Schmittgen T.D. (2001). Analysis of relative gene expression data using real-time quantitative PCR and the 2^−ΔΔCT^ Method. Methods.

[52] Rao X., Huang X., Zhou Z., Lin X. (2013). An improvement of the 2^−ΔΔCT^ method for quantitative real-time polymerase chain reaction data analysis. Biostat. Bioinform. Biomath..

